# Deeper understanding of mechanisms contributing to sepsis-induced myocardial dysfunction

**DOI:** 10.1186/cc13853

**Published:** 2014-05-01

**Authors:** Keith R Walley

**Affiliations:** 1 Centre for Heart Lung Innovation, St Paul’s Hospital, University of British Columbia, Vancouver, BC V6Z 1Y6, Canada

## Abstract

The inflammatory response of sepsis results in organ dysfunction, including myocardial dysfunction. Myocardial dysfunction is particularly important in patients with severe septic shock who progress to a hypodynamic pre-terminal phase. Multiple aspects of this septic inflammatory response contribute to the pathogenesis of decreased ventricular contractility. Inflammatory cytokines released by inflammatory cells contribute as does nitric oxide released by vascular endothelium and by cardiomyocytes. Endotoxins and other pathogen molecules induce an intramyocardial inflammatory response by binding Toll-like receptors on cardiomyocytes that then signal via NF-κB. These processes alter cardiomyocyte depolarization and, therefore, contractility. The particular role of the cardiomyocyte sodium current has not been characterized. Now new information suggests that the septic inflammatory response impairs normal depolarization by altering the cardiomyocyte sodium current. This results in decreased ventricular contractility. This is important because new targets for therapeutic intervention can be considered and new approaches to evaluation of this problem can be contemplated.

## 

In the previous issue of *Critical Care*, Koesters and colleagues [1] contribute to our understanding of sepsis-induced ventricular dysfunction. Sepsis-induced ventricular dysfunction is increasingly recognized as a contributor to cardiovascular dysfunction during septic shock [2]. Vasodilation induced by nitric oxide (NO) and other mediators figures prominently in the hypotensive distributive shock circulation induced by sepsis. But additional sepsis-induced ventricular dysfunction can result in an inadequate cardiac output response to decreased afterload, resulting in pre-terminal hypodynamic septic shock [3]. It could be argued that decreased ventricular contractility during sepsis is not a major issue in hyperdynamic septic shock characterized by an elevated cardiac output. Yet death due to septic shock is characterized by onset and progression of a hypodynamic phase that, in the volume-resuscitated patient, can only be explained by myocardial dysfunction. So ventricular dysfunction may be less important in septic shock survivors but myocardial dysfunction of sepsis may well be the deciding factor in early mortality of septic shock.

The septic inflammatory response involves a surprising number of intersecting pathways, many of which contribute to ventricular dysfunction. Pathogen-associated molecular patterns (PAMPs) released by infecting organisms bind innate immune receptors on inflammatory cells (for example, monocytes, neutrophils) but also bind innate immune receptors in the heart [4] and within the coronary macro- and micro-circulation. For example, cardiomyocytes express a variety of Toll-like receptors that, when bound by a PAMP, induce intracellular signaling via NF-κB, which then induces an intra-myocardial inflammatory response [5]. In addition, early pro-inflammatory cytokines, such as tumor necrosis factor-α and interleukin-1β [6], released by similarly activated inflammatory cells contribute in part by increased production of NO [7]. NO can decrease ventricular contractility by activating guanylate cyclase, which results in decreased intracellular calcium concentration. A variety of inflammatory mediators induce expression of cellular adhesion molecules by endothelial cells of the coronary microcirculation and even by cardiomyocytes. Ligation of cardiomyocyte-expressed intracellular adhesion molecule (ICAM)-1 by ligands such as fibrinogen result in decreased contractility [8]. Chemotactic cytokines such as macrophage inflammatory protein 2 are also expressed by activated cardiomyocytes. Increased production of chemokines leads to retention of neutrophils and other leukocytes in the coronary circulation and extravasation of these inflammatory cells into the myocardium. Contact between leukocytes and cardiomyocytes mediated by ICAM-1 and other adhesion molecules also results in a decrease in cardiomyocyte contractility [9]. Ligation of cardiomyocyte-expressed ICAM-1 signals via the cortical actin cytoskeleton resulting in altered intracellular calcium flux [10].

Alteration of intracellular calcium flux is a fundamental later step in the mechanistic pathway to decreased cardiomyocyte and ventricular contractility. In the previous issue of *Critical Care* Koesters, Engisch and Rich investigate the role of cardiomyocyte sodium current [1]. Alterations in sodium current have been observed in skeletal muscle and in nerves so these authors tested the hypothesis that similar abnormalities may be observed in myocardial tissue. Papillary muscles excised from septic rats 1 day after cecal ligation and puncture were studied. They found that action potentials of papillary muscles from septic rats differed substantially from those of controls. Action potential magnitude was decreased, the threshold was increased, and the rate of rise was decreased. Notably, the threshold and rate of rise of the action potential are known to be heavily influenced by the sodium channel. To put these results into context, these investigators compared these intra-abdominal sepsis observations to those where the sodium channel was inhibited using tetrodotoxin. There were similarities between sepsis-induced changes and tetrodotoxin-induced changes. Both intra-abdominal sepsis and a comparable concentration of tetrodotoxin (similar effects on the action potential) decreased papillary muscle force by more than half. The authors conclude that the sepsis-induced changes in sodium channel function could contribute substantially to decreased ventricular contractility during sepsis.

To determine whether other channel currents could have contributed, additional characteristics of the septic action potential were measured. There was no significant change in resting potential, a property influenced by non-voltage gated K channels. Membrane resistance and action potential duration decreased only slightly in septic action potentials, suggesting a limited role, at best, for voltage gated K and Ca channels. Inhibition of the L-type Ca current reduced contractility, as expected.

Thus, these novel results raise the possibility that sepsis-induced decreases in sodium channel flux may contribute to ventricular dysfunction during sepsis. This conjecture is based on similarity between sepsis-induced and tetrodotoxin-induced changes and therefore is not mechanistic proof of a role of the sodium channel. Nevertheless, moving the focus of investigation towards intracellular signaling expands our knowledge of potential mechanistic pathways contributing to myocardial dysfunction of sepsis. This is important because new targets for therapeutic intervention can be considered and new approaches to evaluation of this problem can be contemplated, possibly even based on surface measurements of electrical properties of the heart.

## Abbreviations

ICAM: Intracellular adhesion molecule; NF-κB: Nuclear factor kappa B; NO: Nitric oxide; PAMP: Pathogen-associated molecular pattern.

## Competing interests

The author declares that he has no competing interests.
